# A Simple Derivation of the Distribution of Pairwise Local Protein Sequence Alignment Scores

**Published:** 2008-02-14

**Authors:** Olivier Bastien

**Affiliations:** CNRS (UMR 5168) - INRA (UMR 1200) - CEA - Université Joseph Fourier, Laboratoire de Physiologie Cellulaire Végétale; CEA Grenoble, 17 rue des Martyrs, F-38054, Grenoble cedex 09, France

**Keywords:** conservation function, reliability theory, Karlin-Altshul theorem

## Abstract

Confidence in pairwise alignments of biological sequences, obtained by various methods such as Blast or Smith-Waterman, is critical for automatic analyses of genomic data. In the asymptotic limit of long sequences, the Karlin-Altschul model computes a *P-value* assuming that the number of high scoring matching regions above a threshold is Poisson distributed. Using a simple approach combined with recent results in reliability theory, we demonstrate here that the Karlin-Altshul model can be derived with no reference to the extreme events theory.

Sequences were considered as systems in which components are amino acids and having a high redundancy of Information reflected by their alignment scores. Evolution of the information shared between aligned components determined the Shared Amount of Information (SA.I.) between sequences, i.e. the score. The Gumbel distribution parameters of aligned sequences scores find here some theoretical rationale. The first is the Hazard Rate of the distribution of scores between residues and the second is the probability that two aligned residues do not lose bits of information (i.e. conserve an initial pairing score) when a mutation occurs.

## Introduction

Almost all sequence alignments methods compute a score s(*a*, *b*) between two compared sequences *a* and *b*. This score is a measure of similarity between the two sequences and help to distinguish biologically significant relationship from chance similarities (Smith and Waterman, 1981; Altschul et al. 1990; Waterman, 1995). These methods assign scores to insertions, deletions and replacements, and compute an alignment of two sequences that corresponds to the least costly set of such mutations. Assignment of a similarity measure begins with a matrix of similarity scores for all possible pairs of residues. Identities and conservative substitution have positive scores, while unlikely replacements have negative scores (Dayhoff et al. 1978; Henikoff and Henikoff, 1992; Bastien et al. 2005a). The score of the computed alignment is the sum of the elementary scores for each pair of aligned residues. All these methods allow the introduction of gaps in the alignment to maximize the final score and to taking account of deletion events in DNA (Waterman, 1995).

Because of the exponential increase of the number of sequence in each database and the large number of sequenced genomes, confidence in alignment score probabilities is critical to perform a rapid and accurate discrimination between alignments. The two main probability models compare the score s(*a*, *b*) with a score computed using random sequence A and B.

The first method proposed by Karlin and Altschul (1990) is an estimate of the probability of an observed local ungapped alignment score according to an Extreme Value Distribution, (or EVD; for review: Coles, 2001) in the asymptotic limit of long sequences. The Karlin-Altschul formula is the consequence of interpreting the number of highest scoring matching regions above a threshold by a Poisson distribution (Karlin and Altschul, 1990). As a consequence, if *s* is the score obtained after aligning two real sequences *a* and *b* (with *m* and *n* their respective lengths), the probability of finding an ungapped segment pair with a score lower than or equal to *s*, follows a particular Gumbel distribution (named EVD type I):

(1) P(S(A,B)≤s)≈exp(-K.m.n.exp(-λ.s))

where *S*(*A*, *B*) is the random variable corresponding to the score of two random sequences, *m* and *n* are the length of the sequences, K and λ are calculated from the scoring matrix and sequence compositions but doesn’t have biological meaning. The *P-value*, defined as the probability of finding an ungapped segment pair with a score higher than *s*, is simply given by 1-*P*(S(*A*, *B*)≤ *s*). Unfortunately, it doesn’t exist similar results for gapped alignment despites numerous theoretical advances (Kschischo et al. 2005). Moreover, the Karlin-Altschul theorem doesn’t apply when the amino acids distribution of the two compared sequences are too dissimilar and when the difference between the lengths of the two sequences is too large. Numerous simulations (Webber and Barton, 2001; Pang et al. 2005) are shown that random score distribution seems to fit, at least the tail distribution, a Standard Gumbel distribution

(2) $$P(S(A,B)\leq s)\approx \exp\left(-\exp\left(-\frac{s-\theta }{\beta }\right)\right)$$

where θ and β are position and scale parameters. All these simulations insist on the fact that Gamma distributions fits better than the equation (1) in the range of low score (bellow s = 30) but it must be notice that the Karlin-Altschul theorem doesn’t hold in this case. As a consequence, the Karlin-Altchul formula is a particular Gumbel distribution where θ and β are given in term of *K*, *m*, *n* and λ Recently, it has been suggest that λ could be interpreted as a parameter of the probability of mutation from one residue to another (see below, Bastien, 2006; Bastien and Marechal, submitted).

The second method uses Monte Carlo simulations to investigate the significance of a score *s* calculated from the alignment of two real sequences *a* and *b* (Lipman and Pearson, 1985; Pearson, 1998; Comet et al. 1999; Bacro and Comet, 2001; Bastien et al. 2004). This is done by shuffling *b* and compute the Z-Value *Z* (*a*, b*) = (*s* − μ̂)/σ̂ with μ̂ the empirical mean score, σ̂ the standard deviation and where * indicates the sequence that was submitted to randomization. Using standard results on the Gumbel distribution, it can be shown that if random scores follow a Standard Gumbel law (Equation (2)), then the Z-Value follow approximately the distribution $P(Z\leq z)=\exp(-\exp(-z\frac{\pi }{\sqrt{6}}-\gamma ))$ (Pearson, 1998; Bastien, 2006), with γ the Euler-Mascheroni constant (γ ≈ 0.5772). This last formula is the exact distribution if and only if we put the exact mean and the exact standard deviation of the score distribution instead of their estimate values. As a consequence, if one can demonstrate that random scores follow a law of type equation (1), one can use the z-value of estimates the wanted probability, ignoring the exact expression of θ and β in terms of *m*, *n* and others unknown parameters. The distribution of Z-Value was observed by Comet et al. (1999) and Weber and Barton (2001) but they didn’t used this formula to fit there data.

From a theoretical point of view, the only thing that can be measured between two biological sequences primary structures is the Shared Amount of Information (SAI) between them. It is well-known that the SAI decreases in average with time because sequences accumulate mutations during this period. The SAI between two sequences can be defined by two ways. The first is to define it by a mathematical distance between the two sequences. Using the Information Theory, it has recently been demonstrate that the Mutual Information I(a; b) between the two sequences *a* and *b* is a better measure of the SAI than all mathematical distances one can compute between *a* and *b* (Bastien et al. 2005b). Indeed, Mutual Information between events *i* and *j* is measured as $I(i;j)=\log\frac{p(i\cap j)}{p(i)p(j)}$ and this is exactly the way to obtain the score between each residues pair, and so the score between each pairs of sequences (Bastien et al. 2005b). Mutual Information between residues is usually computed from known alignments (Henikoff and Henikoff, 1992) or Markov chain process (Dayhoff et al. 1978) and Mutual Information between sequences is the sum of all aligned residues pairs (Bastien et al. 2005b). As a consequence, the score s(*a*, *b*) computed in Sequence Analysis Theory is the estimate of the Mutual Information between the two sequences. In the rest of the text, SAI will identify with Mutual Information MI. Using concept from Reliability Theory (For review: Gavrilov and Gavrilova, 2001), Bastien (2006) demonstrate that, in the asymptotic limit of long sequences or high pairwise score, the Karlin-Altschul formula can be rewritten

(3) ψ(s)=kmn.exp(-λs)

where ψ(*s*) is the Information Conservation Function (C.F.)

(4) $$\psi (s)=\frac{d\ln P(s)}{ds}$$

with *P*(*s*) the Probability of having a score less than s. The λ parameter allows also a simple interpretation, since it is the hazard rate corresponding to the probability for any given residue to align with a residue with a score lower than s bits (for more details, see Bastien, 2006). In detail, the Information C.F. is the probability per units of bits of having a Mutual Information between *a* and *b* of *s*(*a*, *b*), knowing that it is not larger than *s*(*a*, *b*). It must be notice that the C.F. is not a probability measure. In fact, taking the fundamental definition of the cumulative distribution P(s) in terms of the density *f* (*s*) = *dP*(*s*)/*ds* reveals, from the differential definition given by equation (4), that the C.F. is simply f(s) renormalized to take account of the additional information that s is bounded to a smaller interval. One feature of the Conservation Function is the transformation of the product of independent probability into a sum of C.F., that is to say, if ψ*_p_*(*s*) = *d* ln *P_p_*(*s*)/*ds* and if *P*(*s*) *P_p_*(*s*)*^n^*, then

(5) $$\psi (s)=\frac{d\ln P{\left(s\right)}^{n}}{ds}=n\frac{d\ln P_{p}(s)}{ds}=n\psi_{p}(s)$$

This result was obtained by considering a sequence as a system which evolved according to the evolution of his components, i.e. the residues. On the model of what have been done for establish the Gompertz law of human mortality rate (Shkovskii, 2005), we would like to suggest a simple version of derivation of ψ(*s*) based on a naïve understanding of the MI evolution between sequences. We will show that this approach allows a simple interpretation of the two parameters λ and *K*. This approach can be resumed in three points:

- s has a natural quantum. This follows from the fact that evolution of s is due to mutation from one amino acid to another. This last phenomenon is a discrete one. A natural way to model this phenomenon is to use a Poisson distribution for the number of Mutual Information one residue pairs can lost when a mutation of one of the two residues occurs.
- For one given residue pairs, we estimate the C.F. at the point s = 0. This is done by using the probability that no change in s = 0 occurs during some un-specified evolutionary process, given that on average a decrease of η occurs.
- Expansion of η in terms of s then gives the C.F. in terms of s, and hence the cumulative distribution P(s).

## Derivation of the Conservation Function for Sequence Alignments

### Derivation of the C.F. for residues

First, let us consider two sequences *a* and *b* of length *m* and *n* and having a Mutual Information equal to zero, that is to say *s*(*a*, *b*) = 0. So, each aligned pair of residue have an elementary score set up to zero. If one considers that no positive change is possible, then the probability of an *N* bit-decrease of the MI between the two residues is given by a Poisson distribution,

(6) $$P_{r}(N)=\frac{\eta^{N}\exp(-\eta )}{N!}$$

where η is the average loss of bits, when a negative substitution occurs between the two aligned residues. As a consequence, the two sequences will keep a MI of zero only if there is no loss of bits (i.e., no negative substitution), i.e. P(0). η can be defined as a function of s and η (0) = η_0_ is the average loss of bits when the Mutual Information between the two residues is equal to 0. By definition, we have

(7) $$P_{r}(0)=\exp(-\eta_{0})=\psi_{r}(0)$$

Trivially, the Shared Amount of Information can be higher than zero. The larger the MI, the more probable largest negative substitutions might occur. For a small variation of MI, we can state that the average number of negative substitutions slowly increases with Information, that is to say

(8) $$\eta (s)=\eta_{0}+\lambda s$$

After substitution into equation (6), we obtain ψ(*s*) = exp(−η (*s*)) and so

(9) $$\psi_{r}(s)=e^{-\eta_{0}}\exp(-\lambda s)$$

### Derivation of the C.F. for sequences

For two sequences *a* and *b* of length *m* and *n*, we have *mn* possible pairs of residues, which can lead to negative substitution and so *P*(*N*) = *P_r_*(*N*)*^mn^*. Using equation (5) and (9), we can state the following formula for the C.F. for two sequences:

(10) $$\psi (s)=mn\;\psi_{r}(s)=mne^{-\eta_{0}}\exp(-\lambda s)$$

This formula is identical to the C.F. of the Karlin-Altshul formula, where the parameter k is identified to exp(− η_0_), the probability for a residue to have no Negative Substitution, knowing the fact that there is no Positive Substitution.

## Discussion

This interpretation of the parameter k can be checked quantitatively by comparing the magnitude order of k in sequence comparison simulation and the value of exp(−η_0_) obtained from BLOSUM62 (Henikoff and Henikoff, 1992), the most popular substitution matrix. The reason for comparing magnitude order instead of more accurate values comes from the fact that values obtained from BLOSUM62 are rounded to the integer (Henikoff and Henikoff, 1992) and that the subsequent error made on exp(−η_0_) is quite large.

Estimation of the C.F. at one point is made using its definition, that is to say

(11) $$\begin{array}{l} \psi (s)=\frac{d\ln P(s)}{ds}\\ =-\frac{dP(s-ds)}{P(s)ds}\approx \frac{P(s)-P(s-\Delta s)}{P(s)\Delta s} \end{array}$$

with Δ*s* a small variation of s. As a consequence, an estimate of ψ*_r_*(0) is

(12) $$\psi_{r}(0)=\frac{P(0)-P(-1)}{P(0)}$$

where P(0) is the probability for a residue of having a substitution lower or equal to zero. It can easily be checked that this value ranges from10^−1^, if one takes rounded value present in the BLOSUM62 matrix, to 5.10^−2^, if one considers that only one amino acid realizes exactly P(0). This magnitude order, especially the last, corresponds to that computed by Altschul et al. (2001).

Future development of this method includes the exact computation of each residue C.F. and of gap penalty so as to obtain a rapid calculation of the alignment score statistics without pre-computed parameters Altschul et al. (2001) and without expensive simulations (Aude and Louis, 2002; Bastien et al. 2007). Whereas Karlin and Altschul (1990) derived their formula by considering maximum of sums of random elementary scores and obtained an extreme value distribution, we demonstrate here that this formula can arise from a different way considering the elementary SAI shared by residues. The elementary Information losses lead to a global loss of Mutual Information at the sequence level and are characterized by the Gumbel formula (1).
